# Age- and Sex-Associated Pathogenesis of Cell Culture-Passaged Kemerovo Virus in IFNAR^(−/−)^ Mice

**DOI:** 10.3390/ijms25063177

**Published:** 2024-03-09

**Authors:** Camille Victoire Migné, Aurélie Heckmann, Baptiste Monsion, Fauziah Mohd Jaafar, Clémence Galon, Sabine Rakotobe, Lesley Bell-Sakyi, Sara Moutailler, Houssam Attoui

**Affiliations:** 1 UMR1161 VIROLOGIE, INRAE, Ecole Nationale Vétérinaire d’Alfort, ANSES, Université Paris-Est, 94700 Maisons-Alfort, France; camille.migne@anses.fr (C.V.M.); baptiste.monsion@vet-alfort.fr (B.M.); faojaafar@gmail.com (F.M.J.); 2 ANSES, INRAE, Ecole Nationale Vétérinaire d’Alfort, UMR BIPAR, Laboratoire de Santé Animale, 94700 Maisons-Alfort, France; aurelie.heckmann@anses.fr (A.H.); clemence.galon@anses.fr (C.G.); sabine.rakotobe@anses.fr (S.R.); 3 Department of Infection Biology and Microbiomes, Institute of Infection, Veterinary and Ecological Sciences, University of Liverpool, 146 Brownlow Hill, Liverpool L3 5RF, UK; lsakyi@liverpool.ac.uk

**Keywords:** Kemerovo virus, serial and alternated passages, mammalian cells, tick cell line, age- and sex-related bias, virulence

## Abstract

Kemerovo virus (KEMV) is a tick-borne orbivirus transmitted by ticks of the genus *Ixodes*. Previous animal experimentation studies with orbiviruses, in particular the interferon receptor double knock-out (IFNAR^(−/−)^) mouse model, did not indicate bias that is related to age or sex. We endeavoured to assess the effect of serial and alternated passages of KEMV in mammalian or *Ixodes* cells on virus replication and potential virulence in male or female IFNAR^(−/−)^ mice, with important age differences: younger males (4–5 months old), older males (14–15 months old), and old females (14–15 months old). After 30 serial passages in mammalian or tick cells, or alternated passages in the two cell types, older female mice which were inoculated with the resulting virus strains were the first to show clinical signs and die. Younger males behaved differently from older males whether they were inoculated with the parental strain of KEMV or with any of the cell culture-passaged strains. The groups of male and female mice inoculated with the mammalian cell culture-adapted KEMV showed the lowest viraemia. While older female and younger male mice died by day 6 post-inoculation, surprisingly, the older males survived until the end of the experiment, which lasted 10 days. RNA extracted from blood and organs of the various mice was tested by probe-based KEMV real-time RT-PCR. Ct values of the RNA extracts were comparable between older females and younger males, while the values for older males were >5 Ct units higher for the various organs, indicating lower levels of replication. It is noteworthy that the hearts of the old males were the only organs that were negative for KEMV RNA. These results suggest, for the first time, an intriguing age- and sex-related bias for an orbivirus in this animal model. Changes in the amino acid sequence of the RNA-dependent RNA polymerase of Kemerovo virus, derived from the first serial passage in *Ixodes* cells (KEMV Ps.IRE1), were identified in the vicinity of the active polymerase site. This finding suggests that selection of a subpopulation of KEMV with better replication fitness in tick cells occurred.

## 1. Introduction

Kemerovo virus (KEMV) is a tick-borne virus which belongs to the species *Great Island virus* within the genus *Orbivirus* (family *Sedoreoviridae*, order *Reovirales*). KEMV was first isolated from adult female *Ixodes persulcatus* collected in 1962 in the Kemerovo region of Russia. The virus was subsequently isolated from human patients with aseptic meningitis [1,2]. Seroconversion studies were conducted in healthy humans residing in the Kemerovo region of western Siberia, and about 2.8% of the tested population had antibodies to KEMV [1,3]. KEMV was also isolated from *Ixodes ricinus* ticks in 1964 in Czechoslovakia [4] and in 1975 in the Vologda region of Russia [3]. KEMV has been incriminated in human cases of encephalitis in Russia and central Europe [5].

Lipovnik virus (LIPV) and Tribec virus (TRBV) are closely related to KEMV, and previous studies indicated that Kemerovo, Lipovnik and Tribec virus are three serotypes of the Kemerovo virus species which should be recognised as a separate species from *Great Island virus* [6]. LIPV was first isolated from adult female *I. ricinus* collected in 1963 in Lipovnik village, Slovakia, whilst TRBV was isolated from nymphs of *I. ricinus* collected in the Tribec mountains in Slovakia. These geographical sites represent natural foci of infection for these viruses [1,7]. Seroprevalence studies in apparently healthy humans residing in western and eastern Slovakia were conducted. It was found that 3% of the surveyed population had antibodies to TRBV (3%), while 18% had antibodies to LIPV. Tribec virus was also isolated from the rodents *Clethrionomys glareolus* (red-back mouse) and *Pitymys subterraneus* (pine mouse) and from goats in Slovakia [1,2,7].

Although KEMV was isolated from *Ixodes* spp. ticks [3,4], field vectors of this virus have not been formally identified. We previously assessed the vector competence of artificially infected *I. ricinus* from France and Slovakia and *I. persulcatus* ticks from Russia for KEMV and showed that it infects French and Slovakian *I. ricinus* and Russian *I. persulcatus* [8]. The infection rates of engorged larvae were comparable for French and Slovakian *I. ricinus* larvae and nymphs and *I. persulcatus* larvae. The rates of trans-stadial transmission were consistently higher in Slovakian *I. ricinus* and *I. persulcatus* [8]. French and Slovakian *I. ricinus* ticks which were infected at the larval stage became negative when tested by real-time RT-PCR four months post-moulting [8]. In contrast, *I. ricinus* from Slovakia and *I. persulcatus* from Russia infected at the nymphal stage all remained positive for KEMV as post-moulting adult ticks. These studies indicated that cell lines derived from *I. ricinus* could be used as in vitro laboratory models for KEMV replication in tick vectors.

Orbiviruses have a wide host range and experimental studies pertaining to pathogenesis, antiviral strategies and vaccinology have been conducted in natural hosts and small animal models. IFNAR^(−/−)^ mice have previously been used as a model for studies of the protection induced by experimental orbivirus vaccines or potential antiviral molecules [9,10,11,12]. Orbiviruses, including *Bluetongue virus* (BTV) and *African horse sickness virus* (AHSV), lethally infect these mice which die between days 4 and 5 post-infection [9,12].

Although pathogenesis and vaccine studies have been thoroughly documented, host genetic factors which influence the susceptibility to orbivirus infections, in particular age and/or sex bias that is linked to selection of mutations following cell culture passage, have been overlooked. Previous studies involving other pathogens indicate that host X-linked genes could contribute to an immunological advantage for females in several infections [13,14,15]. Males of various species have been found to be more susceptible to infection with pathogens including parasites, fungi, bacteria and viruses. For instance, notifications of tuberculosis cases are almost twice as high in males than females [16].

Hormonal influence negatively affecting development of suppressive immunity in males has been suspected to be driven by testosterone [16]. However, variations in the Y chromosome have been increasingly linked to the augmented susceptibility of males to viral diseases caused by influenza virus A and coxsackievirus B3. Older age has also been linked to more severe pathological outcomes of viral diseases [16].

In this study, we assessed the phenotypes of KEMV that had been serially passaged in mammalian or tick cells or alternated between the two cell types. We observed age- and sex-related bias in virulence and pathogenesis of KEMV between groups of experimentally infected male or female IFNAR^(−/−)^ mice of comparable age.

## 2. Results and Discussion

### 2.1. Minimal Infective Dose of KEMV in IFNAR^(−/−)^ Mice

On day 2 pi, none of the 8–10-week-old mice that were inoculated with the parental KEMV smb1/Vero2/BSR3 in order to determine the minimal infective dose showed any form of clinical signs. Throughout the experiment, mice that were inoculated with doses between 0.1 and 10^2^ PFU showed no clinical signs or viraemia until the end of the experiment. The mice that were inoculated with 10^3^ and 10^4^ PFU showed viraemia on day 2 pi and clinical signs on day 3 pi. The symptoms included prostration, eyelids permanently shut, drooping ears and haemorrhage from the eyes, nose, mouth, fingers and toes. Upon dissection, we consistently observed hepatomegaly with discoloured livers and splenomegaly with spotty/yellowish spleens. The minimal infective dose was determined to be 10^3^ PFU/mouse.

### 2.2. Virulence of KEMV Passaged in Mammalian or Tick Cells or by Alternation between the Two Cell Systems

**Figure 1:**
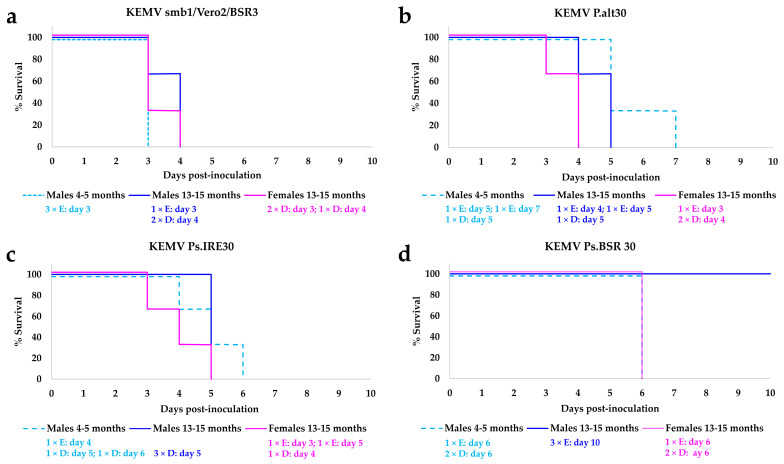
Survival of IFNAR^(−/−)^ mice inoculated with 10^3^ PFU of (**a**) parental Kemerovo virus (KEMV) smb1/Vero2/BSR3, (**b**) KEMV P.alt30, (**c**) KEMV Ps.IRE30 and (**d**) KEMV Ps.BSR30. Three mice inoculated per virus and per age/sex group. In each group, the numbers of animals which were euthanised when the specified endpoint was reached are shown and are identified by the capital letter E, while those which died are identified by the capital letter D.

The phenotype of each passage 30 of KEMV in BSR cells (Ps.BSR30), IRE/CTVM20 cells (Ps.IRE30), or resulting from alternation between BSR and IRE/CTVM20 (P.alt30) and the parental KEMV smb1/Vero2/BSR3, was assessed by subcutaneously inoculating 10^3^ PFU into each of three IFNAR^(−/−)^ mice. Mouse survival was recorded over a period of 10 days, and the survival curves are shown in Figure 1.

Mice that were inoculated with the parental KEMV (KEMV smb1/Vero2/BSR3) all died between days 3 and 4 pi (Figure 1a) with severe clinical signs, regardless of the age or sex of the animals, and with Ct values ranging from 24.78 to 29.16 for KEMV RNAemia.

In mice that were infected with KEMV grown by alternation between tick and BSR cells (P.alt30), females (13–15 months old) were the first to show clinical signs (all three showed clinical signs on day 2 pi), and they died between days 3 and 4 pi (Figure 1b), with Ct values of 28.45–30.57 for KEMV RNAemia. Older males (13–15 months old) all died between days 4 and 5 pi, with Ct values of 29.21–34.86, while the younger males (4–5 months old) died between days 5 and 7 pi, with Ct values ranging from 28.61 to 31.62. The females showed more severe clinical signs, followed by older males then younger males, and this is reflected in the survival curves in Figure 1. These results show similarities to experimental infection studies of mice with St Louis encephalitis virus in which older mice showed the most severe clinical signs [17].

Previously published studies conducted with BTV-17 indicated that the virus sequence remained stable regardless of whether the virus was serially passaged in a mammalian cell line (bovine pulmonary artery endothelial cells, BPAEC), a *Culicoides sonorensis*-derived cell line (uVaW3 cells), or by alternation between the two types of cells. Only minor changes (0.2%) were observed in genome segments (Seg-5 and Seg-10) encoding the non-structural proteins involved in cell exit [18]. However, studies conducted with BTV-3 showed a greater genetic diversity after passage in a different *C. sonorensis*-derived cell line (KC cells), while a subsequent passage in BSR cells significantly narrowed the genetic diversity, with mutations occurring in both structural and non-structural proteins. These mutations were linked to the attenuation of virulence and host specialisation [19].

In mice that were inoculated with KEMV serially passaged in tick cells (Ps.IRE30), old females were again the first to show clinical signs and died between days 3 and 5 pi (Figure 1c), with Ct values ranging from 29.33 to 36.23. The older males all died on day 5 pi, while the younger males died between days 4 and 6 pi, with less variation in Ct values (31.61 to 34.11).

The results obtained with mice which were inoculated with KEMV serially passaged in BSR cells (Ps.BSR30) were intriguing. The younger males and old females all died on day 6 pi (Figure 1d), with Ct values ranging from 34.43 to 35.29 for the younger males and 35.11 to 36.6 for the females. The females were the first to show clinical signs on day 4 pi. However, though older males showed clinical signs by day 6 pi, they all survived until the end of the experiment, and clinical signs were no longer observed by day 8–9. There was no difference in the range of Ct values for RNAemia between younger males, old females and older males. It is noteworthy that on day 10 pi, one older male was found to be non-viraemic as tested by real-time RT-PCR, while the other two had Ct values of 36.77 and 37.28. Serial passage of BTV in mammalian cells has been previously reported to attenuate virulence, and the standard OBP (Onderstepoort Biological Products) vaccines for BTV have been produced by passage in mammalian cell cultures [20]. It appears that the serial passage of KEMV in BSR cells may have led to an attenuation of virulence, as shown by the higher Ct values of KEMV RNAemia.

### 2.3. Replication of KEMV in Mouse Organs

**Figure 2:**
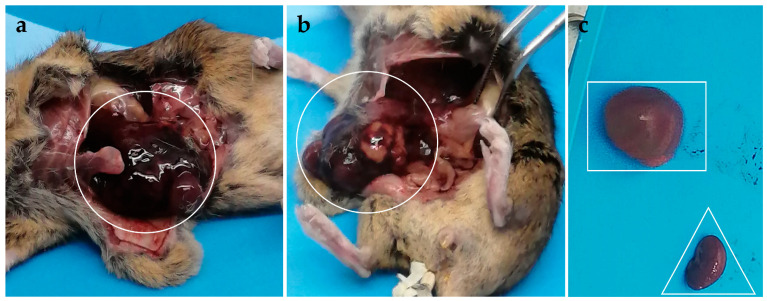
Hepatomegaly in a mouse inoculated with Kemerovo virus (KEMV) Ps.IRE30 ((**a**,**b**), the liver is indicated by a circle) and nephromegaly in a mouse inoculated with parental KEMV smb1/Vero2/BSR3 ((**c**), the enlarged right kidney is indicated by a rectangle and the normal sized left kidney by a triangle).

The heart, brain, lungs and kidneys of all the dissected mice had normal appearance except for one younger male inoculated with parental KEMV smb1/Vero2/BSR3, which showed nephromegaly of the right kidney (Figure 2c). The spleen and liver showed obvious changes, as detailed in Table 1 and shown in Figure 2.

During replication of dsRNA viruses, large quantities of ssRNA are transcribed by virus cores, and only a fraction of these ssRNAs are encapsidated and converted into dsRNA in the nascent cores [21]. Real-time RT-PCR detects viral ssRNA and dsRNA. The *post mortem* changes would likely affect the integrity of viral ssRNA and possibly dsRNA, making quantification of virus replication unreliable. Therefore, when mice died during the night before being observed the next morning, we dissected the animals for the simple observation of morphological changes in the organs but did not collect these organs for RNA extraction and subsequent quantification by real-time RT-PCR. The numbers of euthanised animals that were dissected to collect organs for RT-PCR are reported in Table 2, Table 3, Table 4 and Table 5.

KEMV RNA was detected, by real-time RT-PCR in various organs of mice inoculated with mammalian cell culture-passaged KEMV (Ps.BSR30), as indicated in Table 2. The table also indicates the number of mice dissected for assessing virus replication. Mean Ct values are shown in Table 2 and compared with the Ct values of blood samples.

Because only older IFNAR^(−/−)^ males inoculated with KEMV Ps.BSR30 survived the infection, we tested various internal organs of these mice harvested on day 10 pi by real-time RT-PCR and compared the results with those of internal organs from old females and younger males harvested at earlier time points. Generally speaking, lower Ct values were detected in the RNA extracts of the organs of older males as compared with old females and younger males. More importantly, the hearts of older males inoculated with KEMV Ps.BSR30 were negative for KEMV RNA.

We also compared the Ct values of RNA extracts from organs or blood of mice inoculated with BSR-passaged KEMV (Table 2) with those detected in RNA extracts of organs and blood samples collected from mice inoculated with parental KEMV smb1/Vero2/BSR3 (Table 3). Old females inoculated with the parental virus all died during the night before being observed, and hence their internal organs were only inspected visually. Older males and younger males inoculated with the parental virus had consistently lower Ct values when the same organs were compared with those of the respective groups inoculated with the BSR-passaged KEMV Ps.BSR30.

The results for older males inoculated with KEMV Ps.BSR30 showed the most striking differences, with values > 10 Ct higher (Table 2) than those seen in the mice inoculated with parental KEMV smb1/Vero2/BSR3 (Table 3). Although RNAemia was comparable in the three groups of mice inoculated with KEMV Ps.BSR30, the real-time RT-PCR results from organ samples indicated that in almost every organ tested, there were >5 Ct units of difference between older males on one hand and old females and younger males on the other hand, except for the brains, which showed comparable Ct values. Globally speaking, the absence of detectable KEMV RNA in the older males’ hearts and the higher Ct values in the other tested organs of these older males strongly suggest an intriguing lower susceptibility of these animals. We have previously shown that attenuation of virulence of a BTV generated by reverse genetics correlated with lower levels of replication in IFNAR^(−/−)^ mice, as observed by higher Ct values, regardless of the sex of the mice [22]. The Ct values of mice inoculated with KEMV Ps.BSR30 clearly indicate that there was an attenuation of virulence. Virus virulence usually correlates with replication fitness [23].

The results of real-time RT-PCR from the organs indicate that the different groups of mice inoculated with KEMV grown by alternation between tick and BSR cells (P.alt30) had broadly comparable Ct values for KEMV RNA (Table 2). These values were 4–6 Ct higher than those detected in the same organs of mice inoculated with the parental KEMV smb1/Vero2/BSR3 (Table 3). These results again indicate that the thirty alternated passages in the tick and mammalian cells had an impact, reducing KEMV replication fitness. This would not be expected to occur during the natural cycles of transmission between the vector tick and the host(s) as it may constrain virus persistence in the field. A thorough investigation by deep sequencing of the full-length KEMV genome in the RNA extracts of mouse organs will be undertaken in order to explore the composition of the quasispecies.

Among the mice inoculated with KEMV that had been serially passaged in tick cells (Ps.IRE30), organs were collected only from old females and young males for real-time RT-PCR. This is because all the old males died the night before being observed and hence were not euthanised upon reaching the specified end point. The real-time RT-PCR results from the organs of old females and young males had comparable Ct values for KEMV RNA (Table 5). These values were 4–8 Ct higher than those detected in the same organs of mice inoculated with the parental KEMV smb1/Vero2/BSR3 (Table 3). These results indicate that the thirty serial passages in the tick cells appear also to impact KEMV replication fitness.

Hence, regardless of the cell culture regime used, the process of thirty passages in vitro appears to result in a reduction in KEMV replication fitness in mice.

During serial passage of KEMV in BSR cells, we observed an alteration in the capacity of virus to lyse these cells. During the earlier passages, BSR cells were fully lysed within 5–6 days pi and only cell debris was observed by day 6. As the number of passages progressed, cell lysis was noticeably reduced, and by passage 20, it was necessary to lyse the cells mechanically by Dounce homogenisation prior to treating them with Vertrel XF. The virus titres of the parental and the BSR serially passaged KEMV were comparable throughout the passages, and the quantities of genomic dsRNA produced were also comparable, as shown in Figure S1. However, despite the comparable virus titres, serial passage in BSR cells affected both the virus’s capacity to lyse the cells and virus replication fitness in the IFNAR^(−/−)^ mice. By comparison, serial passage of KEMV in tick cells (Ps.IRE30) did not affect cell morphology, and the tick cells remained intact throughout the passage history from passage 1 to passage 30. However, the replication fitness of KEMV Ps.IRE30 in IFNAR^(−/−)^ was also reduced compared with the parental virus. In contrast, KEMV P.alt30 consistently lysed BSR cells as efficiently as the parental KEMV smb1/Vero2/BSR3.

### 2.4. Sequencing of the Parental KEMV smb1/Vero2/BSR3 and KEMV Ps.IRE1

We sequenced the full-length genome of KEMV smb1/Vero2/BSR3 (GenBank accession numbers PP278971-PP278980) and found it to be 17,874 bp long. Genome segments 1–3 and 6–9 of KEMV Ps.IRE1 were also sequenced (GenBank accession numbers PP278981–PP278987). The length of each genome segment, the encoded proteins and the sequence of conserved termini are shown in Table S1.

Sequence comparisons reveal that the nucleotide sequences of KEMV Seg-2, Seg-7 and Seg-9 of smb1/Vero2/BSR3 were 100% identical to those of Ps.IRE1.

The nucleotide sequences of Seg-1 of KEMV smb1/Vero2/BSR3 and Ps.IRE1 were 99.8% identical, with only eight nucleotide changes, which were all transitions. However, three of these changes were synonymous and did not incur amino acid (AA) changes, while the remaining five nucleotide changes were non-synonymous, and each resulted in an AA change (K612R, S662P, R769H, A864V, R943G) in the vicinity of the RNA-dependent RNA-polymerase (RdRp) active site (Figure S2). The motifs of the polymerase active site in Figure S2 are named as previously described [24,25,26].

The nucleotide sequences of Seg-3 of KEMV smb1/Vero2/BSR3 and Ps.IRE1 were 99.90% identical, with two nucleotide changes, the former being a transversion (T813A) resulting in the AA S270T change and the second being a transition (G1408A) resulting in the AA R468K change. The nucleotide sequences of Seg-6 of KEMV smb1/Vero2/BSR3 and Ps.IRE1 were 99.94% identical, with a single synonymous transversion (A6C). The nucleotide sequences of Seg-8 of KEMV smb1/Vero2/BSR3 and Ps.IRE1 were 99.91% identical with a single transition (C668T) resulting in the AA A217V change.

## 3. Materials and Methods

### 3.1. Cell Lines, Virus and Mice

BSR cells (a clone of BHK-21, [27]) were grown at 37 °C in Dulbecco’s Modified Eagle Medium (DMEM) supplemented with 10% foetal bovine serum (FBS), 100 IU of penicillin and 100 µg of streptomycin per mL, under 5% CO_2_.

The *I. ricinus* cell line IRE/CTVM20 (Tick Cell Biobank, University of Liverpool, Liverpool, UK) was grown at 30 °C in a specialised medium consisting of equal parts of L-15 and L-15B, prepared as previously described [28].

KEMV was isolated, in 1962, from the brain of a patient who died from an acute encephalitis [6] and was kindly provided as a lyophilised sample by Prof. R. Tesh (University of Texas Medical Branch, Galveston, TX, USA). The strain which we used has been previously grown once in suckling mouse brain (smb), passaged twice in Vero cells and three times in BSR cells. This strain was designated parental KEMV smb1/Vero2/BSR3.

IFNAR^(−/−)^ mice (genetic background: A129SvEvBrd) [29] were a gift from Prof. Michel Aguet (ISREC, Ecole Polytechnique Fédérale de Lausanne, Lausanne, Switzerland). IFNAR^(−/−)^ mice can be lethally infected with orbiviruses, such as BTV (serotypes 1 to 24) [9,11,12,30,31]. Male and female IFNAR^(−/−)^ mice aged 8–10 weeks, 4–5 months or 13–15 months were used in this study. All experimental protocols were approved by ANSES-ENVA-UPEC Ethics Committee for Animal Experimentation (Agreement Number: 19-028).

### 3.2. Virus Release from Cells/Cell Debris and Titration

BSR cells infected with orbiviruses become fully lysed within 5 days post-infection, and parental KEMV smb1/Vero2/BSR3 does not deviate from this characteristic [32,33]. Upon lysis, over 95% of the virus particles remain associated with cell debris. Arthropod cells derived from orbivirus vectors are not lysed post-infection with orbiviruses, and KEMV does not deviate from this characteristic either. Most of the KEMV particles remain within the intact cells.

Vertrel XF (Sigma-Aldrich, Saint-Quentin-Fallavier, France) is an organic solvent that facilitates freeing orbivirus particles from interaction with cell debris [22,34]. On day 6 post-infection, BSR cell lysates, including cell debris and culture supernatants, were collected. An equal volume of Vertrel XF was added, and the mixture was shaken vigorously before being centrifuged at 2000× *g* for 10 min at 4 °C. The aqueous phase, which contained the free virus particles, was collected and used for titration.

Infected tick cells were collected on day 6 post-infection using a cell scraper and pelleted by centrifugation at 200× *g* for 5 min at 4 °C. The cell pellet was washed by centrifugation three times in serum-free medium and once in a hypotonic solution (10 mM HEPES, 1.5 mM MgCl_2_ and 10 mM KCl). The cells were suspended in 1.5 mL of the hypotonic solution and left at room temperature for 10 min until cell swelling was obvious by microscopic examination. The suspension was subjected to 10 stokes in a Dounce homogeniser followed by addition of 1 mL of Vertrel XF and further subjected to ten strokes. The suspension was centrifuged at 2000× *g* for 10 min at 4 °C, and the supernatant was collected and diluted with an equal volume of L-15/L-15B medium.

KEMV titre was determined by plaque assay [8]. Cell culture plates (24-well) were seeded with BSR cells (1 × 10^5^ cells/well) the day before titration. Ten-fold serial dilutions of the KEMV suspension were prepared in serum-free DMEM and added to the wells. Following adsorption, the cell monolayers were overlaid with molten 1% low-melting point agarose in DMEM. On day 5 post-infection, the wells were treated with 10% formaldehyde, the cell monolayers were stained with 0.1% naphthalene-black solution and visible plaques were counted.

### 3.3. Passages of KEMV in BSR and/or IRE/CTVM20 Cells

**Figure 3:**
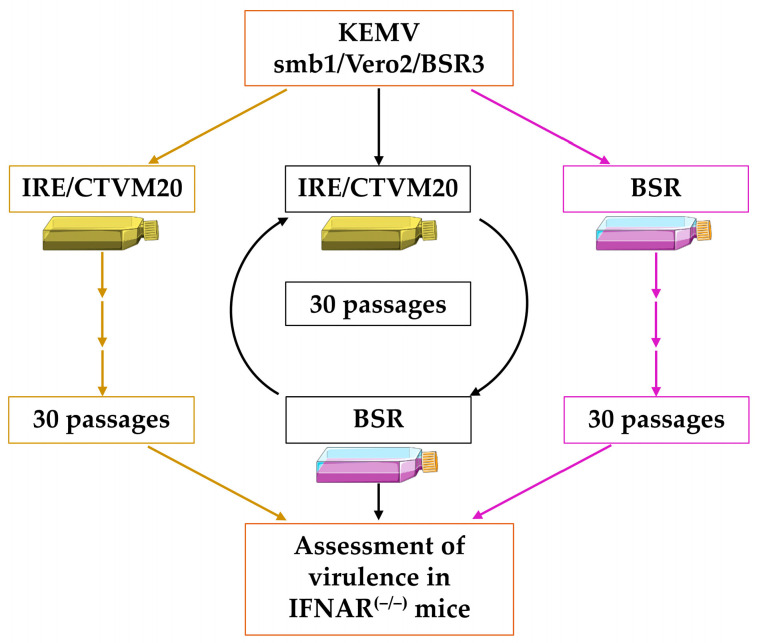
Schematic representation of the cell culture passage strategy of Kemerovo virus (KEMV) in BSR or IRE/CTVM20 cells or by alternation in both cell systems.

The cell culture passage strategy is shown in Figure 3. This strategy is based on previously published studies which assessed replication fitness of arboviruses in mammalian and or arthropod cells, in which a given virus was subjected to 10–30 serial or alternated passages. Parental KEMV smb1/Vero2/BSR3 was subjected to 30 serial or alternated passages in tick and/or mammalian cells. The multiplicity of infection was 0.05 plaque forming units (PFU)/cell. Infections in BSR or IRE/CTVM20 cells lasted 6 days at 37 °C and 30 °C, respectively. Serial passages were designated Ps.IRE1 to 30 (tick cells) and Ps.BSR1 to 30 (BSR cells). Alternated passages in BSR and tick cells were designated P.alt1 to P.alt30, starting with P.alt1 in IRE/CTVM20 cells and ending with P.alt30 in BSR cells.

### 3.4. Determination of the Minimal Infective Dose of KEMV in IFNAR^(−/−)^ Mice

The minimal infective dose of KEMV was determined by subcutaneous (SC) inoculation of 8–10-week-old IFNAR^(−/−)^ mice with 100 µL/mouse of diluted virus suspensions. The inoculated suspensions contained between 0.1 and 1 × 10^4^ PFU/100 µL of parental KEMV smb1/Vero2/BSR3 (Table 6). Each suspension was tested in duplicate. Blood samples were collected on days 0, 2, 3 and 5 post infection (pi) in order to assess virus RNAemia by real-time RT-PCR and mice were euthanised on day 6. Details of the IFNAR^(−/−)^ mice are shown in Table 6. Based on previous reports, the sex of recipient animals is not known to influence the outcome of infection with orbiviruses, such as BTV or AHSV [29,35].

### 3.5. Assessing Virus Phenotype in IFNAR^(−/−)^ Mice

Previously we have not observed any age- or sex-related bias with other orbiviruses such as bluetongue virus (BTV) or African horse sickness virus (AHSV) [29,35]. In addition, regardless of whether 3, 5 or 6 animals were used, the outcome of infection in IFNAR^(−/−)^ mice, with a specific BTV or AHSV strain/cell culture passage, was identical, and all animals died within the range of 3–5 days. Therefore, to assess the phenotypes of the various passages of KEMV, we used groups each comprising 3 mice: 4–5-month-old males (designated in the study as younger males), 13–15-month-old males (designated as older males), and 13–15-month-old females (designated as old females). No younger females were available for inclusion at the time the study was performed as they were retained in the laboratory colony for breeding purposes.

The phenotype of KEMV resulting from each passage 30 (Ps.IRE30, Ps.BSR30 and P.alt30), as well as that of the parental KEMV smb1/Vero2/BSR3, were assessed following SC inoculation of IFNAR^(−/−)^ mice with the minimal infective dose. Survival was monitored over 10 days, and mice were observed twice daily (morning and evening). Unless they died in the night, mice were euthanised when the specified end point was reached (prostration, inactivity, lacrimation, scruffy fur). In addition to blood, organs were sampled from the euthanised mice (liver, spleen, kidneys, lungs, heart and brain). RNA was extracted from blood and organs and tested by real-time RT-PCR.

### 3.6. Detecting Viral RNA in Mouse Samples

A fraction of each organ (about ¼ liver, ½ spleen, ½ kidney, ½ lung, ½ heart and ½ brain) was homogenised in 500 µL of DMEM supplemented with 10% FBS using six stainless-steel beads in a Precellys^®^24 Dual homogeniser at 5500 rpm for 20 s. RNA from blood and organs was extracted using a NucleoSpin^®^ RNA extract II kit (Macherey Nagel, Düren, Germany) as recommended by the manufacturer. RNA samples were eluted in 40 µL of RNase-free water.

The genomic dsRNA of KEMV was denatured by heating at 99 °C for 5 min and then immediately stored on wet ice for 5 min. Real-time RT-PCR was performed using a LightCycler^®^ 480 RNA Master Hydrolysis kit (Roche Diagnostics, Meylan, France) as recommended by the manufacturer in the presence of Seg-2 derived primers and probes (KEMV_F: 5′-GTCAGACGGATTTTCGACCTC-3′, KEMV_R: 5′-GCGAGCCAGATCCCGATGT-3′ and KEMV_Probe: 5′FAM-ACGGGCCAACACTCGTTCATCACAG-BHQ1 3′) [36]. The PCR cycling parameters were as follows: one cycle of denaturation at 95 °C for 30 s, 45 successive cycles at 95 °C for 10 s, 60 °C for 30 s, and 72 °C for 1 s.

### 3.7. Sequencing of the Parental KEMV smb1/Vero2/BSR3 and KEMV Ps.IRE1 Genomes

The genome segments of the parental KEMV (smb1/Vero2/BSR3) and the first-passaged KEMV in IRE/CTVM20 cells (KEMV Ps.IRE1) were copied into cDNA and sequenced using a single-primer amplification technique as reported previously [37]. Briefly, a defined 3’-amino-blocked DNA oligonucleotide was ligated to both 3’ ends of the dsRNA segments using T4 RNA ligase (New England Biolabs, Ipswich, MA, USA) followed by reverse transcription and PCR amplification using a complementary primer. The PCR amplicons were analysed and purified by agarose-gel electrophoresis, ligated into the pGEM-T cloning vector (Promega, Madison, WI, USA) and used to transform chemically competent XL1-Blue *E. coli* (Agilent Technologies, Les Ulis, France). Plasmids were purified using the Qiaprep Miniprep kit (Qiagen, Les Ulis, France) and 3 plasmid clones of each genome segment were subjected to Sanger sequencing.

The sequences of the genome segments of KEMV smb1/Vero2/BSR3 and Ps.IRE1 were aligned using ClustalX software (version 2.1) [38] and analysed using Mega11 software (version 11.0.10) [39].

## 4. Conclusions

The observed age/sex-related bias in IFNAR^(−/−)^ mice inoculated with cell culture-passaged KEMV has not been previously reported for other orbiviruses in this animal model. Old female IFNAR^(−/−)^ mice exhibited a heightened sensitivity to the cell culture-passaged KEMV (after 30 passages) regardless of the cell system which we used.

Although females are less affected by infectious diseases in terms of prevalence, as mentioned above in the Introduction [13,14,15,16], it has been reported that older females infected with arboviruses such as dengue, Zika or chikungunya virus exhibited higher rates of more severe clinical symptoms [40]. Furthermore, mice inoculated with St. Louis encephalitis virus showed an age-related bias, with older mice showing the most severe clinical signs [17]. However, the observations obtained for the old males (13–15 months old) inoculated with KEMV which was serially passaged in BSR cells remain the most intriguing finding, and warrant further investigations, including genetic linkage analyses in relation to the adaptation to mammalian cell culture. These mice remained infected and survived until the end of the experiment (day 10 pi).

Genome sequencing of the parental strain of KEMV made comparisons possible with the genome sequences derived from various cell culture passages and with those of the specific subpopulations of KEMV in various mouse organs.

Comparison with the sequence of the first passage in tick cells revealed nucleotide changes in four out of the seven sequenced genome segments. Both synonymous and non-synonymous changes were observed. The most notable non-synonymous changes incurred amino acid changes that were observed in genome segment 1 (encoding the viral RNA-dependent RNA polymerase) in the vicinity of the polymerase active site. This finding likely reflects the selection of a subpopulation of KEMV with a better replication fitness in tick cells.

These findings highlight the importance of assessing the diversity of the various viral populations, both after cell culture passage and in mouse organs, to clarify the full extent of underlying reasons for the observed age- and sex-related bias. Deep sequencing of the full length KEMV genomes of these various samples will be undertaken. This will help in assessing the effect of passages in mammalian and/or tick cells on replication fitness and provide insight into the genetic diversity/evolution of these viruses and the process of compartmentalisation of subpopulations from the quasispecies into different organs of the mice. Further investigations will be conducted in order to assess whether this phenomenon is restricted to KEMV or if it could also occur more widely with other tick-borne orbiviruses such as Lipovnik virus and Tribec virus, and these studies will include young and old mice of both sexes.

## Figures and Tables

**Table 1:** Macroscopic observations of spleen, liver and kidneys of IFNAR^(−/−)^ mice inoculated with cell culture-passaged KEMV by subcutaneous route.

Organs	KEMV smb1/Vero2/BSR3	KEMV P.alt30	KEMV Ps.BSR30	KEMV Ps.IRE30
Spleen	No splenomegaly. White/yellow spots.	Splenomegaly. White/yellow spots or entirely yellowish.	Splenomegaly. White/yellow or fully yellowish.	Splenomegaly. White/yellow spots or fully yellowish.
Liver	Slightly enlarged. Discoloured.	Slightly enlarged. Discoloured.	Slightly enlarged. Discoloured. 13–15-month-old males did not show observable changes in their liver.	Hepatomegaly *. Discoloured.
Kidneys	Nephromegaly * (one young male only).	Normal appearance.	Normal appearance.	Normal appearance.

* See Figure 2.

**Table 2:** Detection of KEMV RNA in organs (and comparison with RNAemia) of IFNAR^(−/−)^ mice inoculated with the BSR-passaged KEMV Ps.BSR30.

Organs	Males (13–15 Months) Inoculated with KEMV Ps.BSR30 (*n* = 3)	Females (13–15 Months) Inoculated with KEMV Ps.BSR30 (*n* = 1) *	Males (4–5 Months) Inoculated with KEMV Ps.BSR30 (*n* = 1) *
Liver	+(35.20)	+(28.53)	+(27.94)
Spleen	+(31.14)	+(22.32)	+(23.68)
Lungs	+(35.29)	+(28.03)	+(29.48)
Kidneys	+(36.84)	+(29.86)	+(31.59)
Heart	-	+(32.39)	+(32.05)
Brain	+(37.35)	+(34.72)	+(33.64)
Blood	+(34.84) (3 mice)	+(36.01) (3 mice)	+(34.86) (3 mice)

+: KEMV RNA detected, -: KEMV RNA not detected. Where KEMV RNA was detected, Ct values are indicated in brackets (where more than one mouse survived, the value shown is the mean Ct). The numbers of mice from which organs were collected for RT-PCR are shown in brackets (*n* =). *: the other mice died during the previous night.

**Table 3:** Detection of KEMV RNA in organs (and comparison with RNAemia) of male IFNAR^(−/−)^ mice inoculated with parental KEMV smb1/Vero2/BSR3. Organs were only collected from males, since all females died the night before, and hence their internal organs were only inspected visually.

Organs	Males (13–15 Months) Inoculated with KEMV smb1/Vero2/BSR3 (*n* = 1) *	Males (4–5 Months) Inoculated with KEMV smb1/Vero2/BSR3 (*n* = 3)
Liver	+(21.46)	+(22.73)
Spleen	+(19.08)	+(19.42)
Lungs	+(22.39)	+(22.94)
Kidneys	+(23.25)	+(24.81)
Heart	+(26.15)	+(26.24)
Brain	+(28.41)	+(29.59)
Blood	+(28.76) (3 mice)	+(27.51) (3 mice)

+: KEMV RNA detected, Ct values are indicated in brackets (where more than one mouse survived, the value shown is the mean Ct). The numbers of mice from which organs were collected for RT-PCR are shown in brackets (*n* =). *: the other mice died during the previous night.

**Table 4:** Detection of KEMV RNA in organs (and comparison with RNAemia) of IFNAR^(−/−)^ mice inoculated with the KEMV passaged alternately between BSR and tick cells (P.alt30).

Organs	Males (13–15 Months) Inoculated with KEMV P.alt30 (*n* = 2) *	Females (13–15 Months) Inoculated with KEMV P.alt30 (*n* = 1) *	Males (4–5 Months) Inoculated with KEMV P.alt30 (*n* = 2) *
Liver	+(25.47)	+(28.08)	+(24.59)
Spleen	+(20.53)	+(20.88)	+(21.75)
Lungs	+(28.03)	+(27.12)	+(26.19)
Kidneys	+(28.05)	+(30.05)	+(28.70)
Heart	+(29.53)	+(32.17)	+(30.63)
Brain	+(32.75)	+(33.32)	+(32.02)
Blood	+(31.62) (3 mice)	+(29.26) (3 mice)	+(30.05) (3 mice)

+: KEMV RNA detected. Where KEMV RNA was detected, Ct values are indicated in brackets (where more than one mouse survived, the value shown is the mean Ct). The numbers of mice from which organs were collected for RT-PCR are shown in brackets (*n* =). *: the other mice died during the previous night.

**Table 5:** Detection of KEMV RNA in organs (and comparison with RNAemia) of male IFNAR^(−/−)^ mice inoculated with tick cell-passaged KEMV Ps.IRE30. Organs were only collected from old females and young males, since all old males died the night before and hence their internal organs were only inspected visually.

Organs	Females (13–15 Months) Inoculated with KEMV Ps.IRE30 (*n* = 1) *	Males (4–5 Months) Inoculated with KEMV Ps.IRE30 (*n* = 2) *
Liver	+(29.80)	+(27.35)
Spleen	+(23.74)	+(22.65)
Lungs	+(26.76)	+(29.53)
Kidneys	+(30.93)	+(29.17)
Heart	+(32.31)	+(31.45)
Brain	+(33.73)	+(33.77)
Blood	+(33.16) (3 mice)	+(32.28) (3 mice)

+: KEMV RNA detected. Where KEMV RNA was detected, Ct values are indicated in brackets (where more than one mouse survived, the value shown is the mean Ct). The numbers of mice from which organs were collected for RT-PCR are shown in brackets (*n* =). *: the other mice died during the previous night.

**Table 6:** Details of IFNAR^(−/−)^ mice (8–10-week-old) used to determine the minimal infective dose of parental KEMV smb1/Vero2/BSR3.

Number of PFU	Total Number of Mice	Sex	
		Female	Male
10^4^	2	2	0
10^3^	2	1	1
10^2^	2	1	1
10	2	1	1
1	2	0	2
0.1	2	0	2

## Data Availability

All data are presented in the manuscript.

## Supplementary Materials

The following supporting information can be downloaded at: https://www.mdpi.com/article/10.3390/ijms25063177/s1.
