# Supplementary material for: Age- and Sex-Associated Pathogenesis of Cell Culture-Passaged Kemerovo Virus in IFNAR(−/−) Mice
Source: Int J Mol Sci. 2024 Mar 9;25(6):3177. doi: 10.3390/ijms25063177 (PMC10970428; doi:10.3390/ijms25063177)
Supplement: Supplementary file 1 [file ijms-25-03177-s001.zip › ijms-2805421-supplementary.pdf]

Table S1. Kemerovo virus (KEMV) genome: lengths of dsRNA genome segments, encoded proteins, 5′ and 3′ NCRs, terminal conserved sequences for KEMV smb1/Vero2/BSR3 (a) and KEMV Ps.IRE1 (b).

a)

Segment	Protein	Accession	5' NCR	5′ termini---------3' termini	3' NCR
1: 3896 bp	VP1(Pol); 1285 AA; 145,956 Da	PP278971	11 bp	5'-GUAAAAUGUC---------UAAGGAUAC-3'	28 bp
2: 2792 bp	VP2(T2); 908 AA; 102,750 Da	PP278972	18 bp	5'-GUAAAACUUC---------UAAGGAUAC-3'	47 bp
3: 1934 bp	VP3(Cap); 632 AA; 72,416 Da	PP278973	5 bp	5'-GUAAAAUGUU---------AUAACUUAC-3'	26 bp
4: 1730 bp	VP4(OC1); 554 AA; 62,562 Da	PP278974	17 bp	5'-GUAAAAUCUUA---------CCAAGAUAC-3'	54 bp
5: 1719 bp	NS1(TuP); 529 AA; 60,037 Da	PP278975	39 bp	5'-GUAAAAAUUU---------UUAAGAUAC-3'	96 bp
6: 1667 bp	VP5(OC2); 537 AA; 59,444 Da	PP278976	22 bp	5'-GUAAAAUUUC---------AUAGGUUAC-3'	37 bp
7: 1197 bp	NS2(ViP); 368 AA; 40,934 Da	PP278977	45 bp	5'-GUAAAAUUUU---------AUAAGAUAC-3'	51 bp
8: 1183 bp	VP7(T13); 357AA; 39,479 Da	PP278978	18 bp	5'-GUAAAAUUCU---------AUAAGUUAC-3'	97 bp
9: 1049 bp	VP6(Hel); 317 AA; 34,198 Da<br>NS4; 151 AA; 17,622 Da	PP278979	58 bp	5'-GUAAAAAUUC---------UAAAGAUAC-3'	43 bp
10: 707 bp	NS3; 214 AA; 23,410 Da<br>NS5; 62 AA; 7,743 Da	PP278980	18 bp	5'-GUAAAAUUUC---------AUAGGAUAC-3'	50 bp
			**Consensus**	5'-GUAAAA$^{A}/_{U}{}^{G}/_{U}{}^{U}/_{C}{}^{C}/_{U}$--$^{U}/_{A}{}^{U}/_{A}{}^{A}/_{G}{}^{G}/_{A}{}^{G}/_{C}{}^{A}/_{U}$UAC-3'	

b)

Segment	Protein	Accession	5' NCR	5′ termini---------3' termini	3' NCR
1: 3896 bp	VP1(Pol); 1285 AA; 145,904 Da	PP278981	11 bp	5'-GUAAAAUGUC---------UAAGGAUAC-3'	28 bp
2: 2792 bp	VP2(T2); 908 AA; 102,750 Da	PP278982	18 bp	5'-GUAAAACUUC---------UAAGGAUAC-3'	47 bp
3: 1934 bp	VP3(Cap); 632 AA; 72,402 Da	PP278983	5 bp	5'-GUAAAAUGUU---------AUAACUUAC-3'	26 bp
6: 1667 bp	VP5(OC2); 537 AA; 59,444 Da	PP278984	22 bp	5'-GUAAAAUUUC---------AUAGGUUAC-3'	37 bp
7: 1197 bp	NS2(ViP); 368 AA; 40,934 Da	PP278985	45 bp	5'-GUAAAAUUUU---------AUAAGAUAC-3'	51 bp
8: 1183 bp	VP7(T13); 357AA; 39,507 Da	PP278986	18 bp	5'-GUAAAAUUCU---------AUAAGUUAC-3'	97 bp
9: 1049 bp	VP6(Hel); 317 AA; 34,198 Da<br>NS4; 151 AA; 17,622 Da	PP278987	58 bp	5'-GUAAAAAUUC---------UAAAGAUAC-3'	43 bp
			**Consensus**	5'-GUAAAA$^{A}/_{U}{}^{G}/_{U}{}^{U}/_{C}{}^{C}/_{U}$--$^{U}/_{A}{}^{U}/_{A}{}^{A}/_{G}{}^{G}/_{A}{}^{G}/_{C}{}^{A}/_{U}$UAC-3'	

AA: amino acid, Da: dalton, NCR: non-coding region.

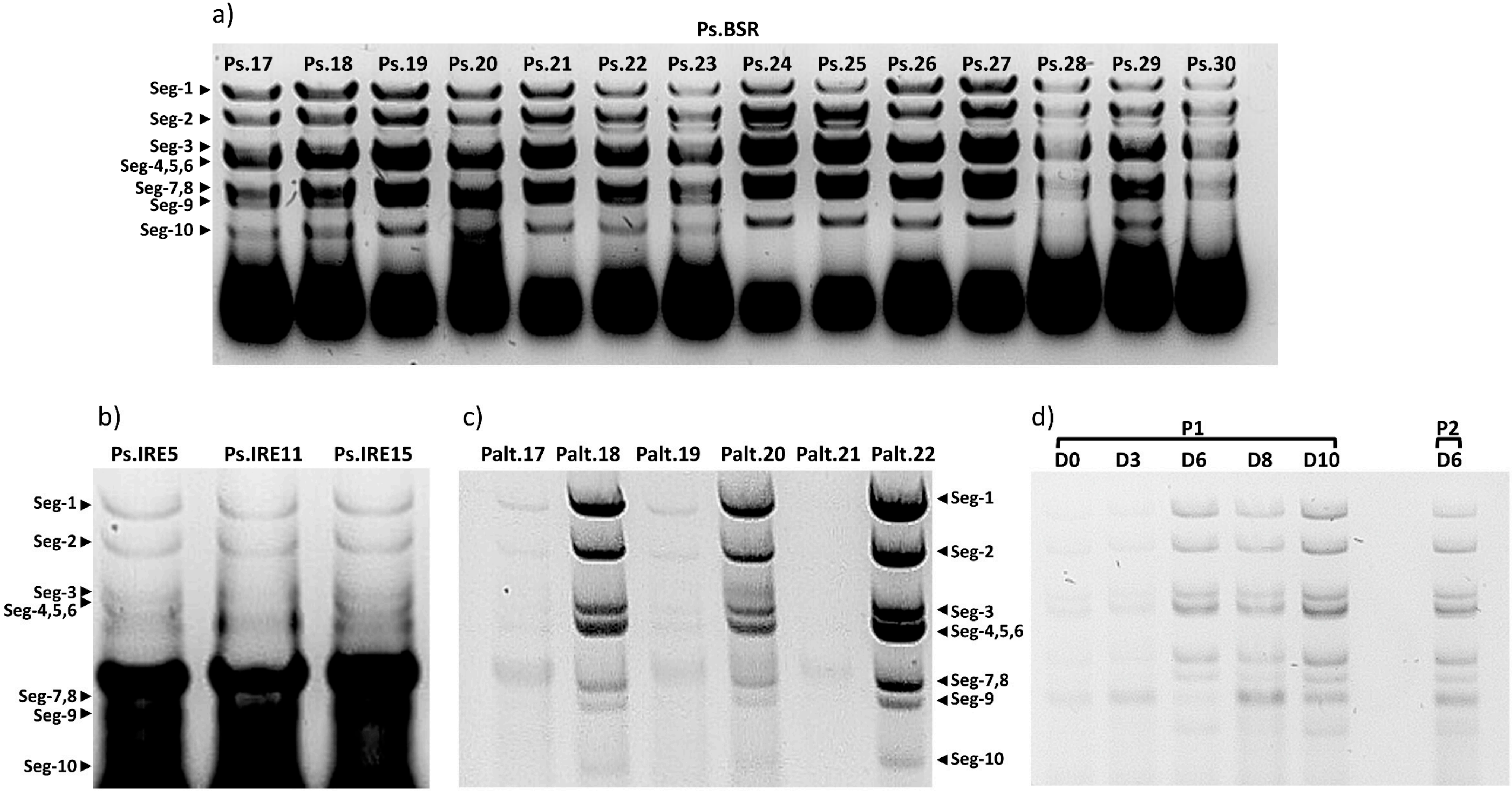

Figure S1. Electropherotypes of the Kemerovo virus (KEMV) dsRNA genome from various cell culture passages. dsRNA extracts were analysed by agarose gel electrophoresis (1% agarose in TAE buffer: 400 mM Tris-Acetate pH 8.0, 10 mM EDTA).

(a) dsRNA genome of KEMV that was serially passaged in BSR cells (Ps.BSR), (b) dsRNA genome of KEMV that was serially passaged in in IRE/CTVM20 (Ps.IRE), (c) dsRNA genome of KEMV that was passaged by alternation between IRE/CTVM20 and BSR cells (P.alt).

(d) This gel shows the growth kinetics of KEMV in IRE/CTVM20. The cells were infected with KEMV and RNA extraction performed from cell pellets on days 0 (D0), 3 (D3), 6 (D6), 8 (D8) and 10 (D10). The maximum levels of dsRNA genome are reached between days 6 and 10. P1: passage 1 in IRE/CTVM20 and P2: passage 2 in IRE/CTVM20.

```
VP1BSR   MAVVEQRITSANNAIRRILPNLPLKDDKTYHDYYKYSKWKDSKATARTTLHGVPVLSENSWRDIPRTTQTRPRD
VP1IRE   MAVVEQRITSANNAIRRILPNLPLKDDKTYHDYYKYSKWKDSKATARTTLHGVPVLSENSWRDIPRTTQTRPRD
         **************************************************************************

VP1BSR   VVEYYYRSQIPVVELQPEEEFLRNYSLDEKDAGPLLAFLRHRSQEEMAVYGDMALRHWYALVQQLSDDYGHAPL
VP1IRE   VVEYYYRSQIPVVELQPEEEFLRNYSLDEKDAGPLLAFLRHRSQEEMAVYGDMALRHWYALVQQLSDDYGHAPL
         **************************************************************************

VP1BSR   GLACMERFIDDHGDPFHQSTRDLSKISDERYSSSVVLFFEMSICEALHEFNVVLRSRESGIPLTTRFGRQEVDR
VP1IRE   GLACMERFIDDHGDPFHQSTRDLSKISDERYSSSVVLFFEMSICEALHEFNVVLRSRESGIPLTTRFGRQEVDR
         **************************************************************************

VP1BSR   IAIVRELYTLMLTHPKKVCNMLRASYSWFVKNWGIAAPEVNLLESSGSDDRNSKDITFHRWRRVRNPYSAIVLA
VP1IRE   IAIVRELYTLMLTHPKKVCNMLRASYSWFVKNWGIAAPEVNLLESSGSDDRNSKDITFHRWRRVRNPYSAIVLA
         **************************************************************************

VP1BSR   TEFHKSSLRANLDKVDEATAYASKLAGTPMELRVFRAMMANTYTTEFDPRDQSHLQLASLLLAIQTMAGYGRAW
VP1IRE   TEFHKSSLRANLDKVDEATAYASKLAGTPMELRVFRAMMANTYTTEFDPRDQSHLQLASLLLAIQTMAGYGRAW
         **************************************************************************

VP1BSR   IVNAGDDPERMLKPSRDNHVTRVARETEKFFVKAYEEARMHGHDIIPPEDMYSSLLRLAKNTSSGFSTEVEVKK
VP1IRE   IVNAGDDPERMLKPSRDNHVTRVARETEKFFVKAYEEARMHGHDIIPPEDMYSSLLRLAKNTSSGFSTEVEVKK
         **************************************************************************

                                                                    Motif I
VP1BSR   KYGPRAERRDEIVRIKSRQKALYLMREGHKIYSPDMMNMSYTTPTCYQTRGTRDVPIKATRTIAINVNVLAPQH
VP1IRE   KYGPRAERRDEIVRIKSRQKALYLMREGHKIYSPDMMNMSYTTPTCYQTRGTRDVPIKATRTIAINVNVLAPQH
         **************************************************************************

                                                                           Motif A
VP1BSR   YILTLPLNEYFARAGGSTHPASSEIGGKIIIGDLEATGSRVMDAADTFRNTSDTGVWTLALDYSNYDTHMTQDN
VP1IRE   YILTLPLNEYFARAGGSTHPASSEIGGKIIIGDLEATGSRVMDAADTFRNTSDTGVWTLALDYSNYDTHMTQDN
         **************************************************************************

VP1BSR   FRRGMISGIRQAVARHHSLKYEGWSVDQLIEAGYGAGRVAGTLWNGKRRVCRMDRADYERLPLEDREVPSDAPF
VP1IRE   FRRGMISGIRQAVARHHSLRYEGWSVDQLIEAGYGAGRVAGTLWNGKRRVCRMDRADYERLPLEDREVPPDAPF
         ******************* *************************************************.****

                                                             Motif B
VP1BSR   RFRPPGTSPIRSLALVKEARTLDFVLVTPWDGSDLARVSTHLSGENSTLVANSLHNMAMGRVIQDEVQTRHPGT
VP1IRE   RFRPPGTSPIRSLALVKEARTLDFVLVTPWDGSDLARVSTHLSGENSTLVANSLHNMAMGRVIQDEVQTRHPGT
         **************************************************************************

                Motif C                             Motif D
VP1BSR   FEVLSEMYVGDDTLHYVKLLTLDSAQIDRAINTIFQTIELCGHEASAAKTTFAPFSAEKTQTHAKQGVYIPQDR
VP1IRE   FEVLSEMYVGDDTLHYVKLLTLDSAQIDHAINTIFQTIELCGHEASAAKTTFAPFSAEKTQTHAKQGVYIPQDR
         **************************** *********************************************

VP1BSR   MMVISSERRKEIEDVQGYMRAQATTFVTKVSRGFSEDLAHRILLLKASLAGYRRFKATIFDGTKFRRRKFFSEE
VP1IRE   MMVISSERRKEIEDVQGYMRAQATTFVTKVSRGFSEDLAHRILLLKASLVGYRRFKATIFDGTKFRRRKFFSEE
         ************************************************* ************************

VP1BSR   DGYTLCRLLNPAVLYAPVECNGYGVHPFALNVVQTCETHLDSIQLFPTYADLVSRNVIRTAFPPPWNETDVDSR
VP1IRE   DGYTLCRLLNPAVLYAPVECNGYGVHPFALNVVQTCETHLDSIQLFPTYADLVSGNVIRTAFPPPWNETDVDSR
         ****************************************************** *******************

VP1BSR   LLSTQTPMGLYSRIVRPTVRMALTDPDIAALVTQIPLGEHAPDRLSSTMMHSALLKESRARALLAPAYEDRFIE
VP1IRE   LLSTQTPMGLYSRIVRPTVRMALTDPDIAALVTQIPLGEHAPDRLSSTMMHSALLKESRARALLAPAYEDRFIE
         **************************************************************************

VP1BSR   SMNKWRPHDFFKPTAGVELTAAYAKVLDLQISARDAPTPLFPDMNLSPAFLAQKQCVGHRTTVRPSRTYMDHID
VP1IRE   SMNKWRPHDFFKPTAGVELTAAYAKVLDLQISARDAPTPLFPDMNLSPAFLAQKQCVGHRTTVRPSRTYMDHID
         **************************************************************************

VP1BSR   RILRGDVVMRGIITANTIMTLLEKIGFDHDPADLAIIFELLNLDRRVARRLAEFVTGDRLRFDVHALNKRGIGG
VP1IRE   RILRGDVVMRGIITANTIMTLLEKIGFDHDPADLAIIFELLNLDRRVARRLAEFVTGDRLRFDVHALNKRGIGG
         **************************************************************************

VP1BSR   DEFTMSLDVCTDGSRDQRITFPPEFTLAERDAAALHAEQIQLLFAAHQGRSVRVALQVRPEHRSALRKARIKMR
VP1IRE   DEFTMSLDVCTDGSRDQRITFPPEFTLAERDAAALHAEQIQLLFAAHQGRSVRVALQVRPEHRSALRKARIKMR
         **************************************************************************

VP1BSR   TPRLRVIRSAARAIRSASLALAEGQFL
VP1IRE   TPRLRVIRSAARAIRSASLALAEGQFL
         ***************************
```

Figure S2: alignment of the amino acid sequences of the RNA-dependent RNA polymerase of parental KEMV smb1/Vero2/BSR3 (VP1BSR) and KEMV Ps.IRE1 (VP1IRE).

The 5 amino acid differences resulting from the 5 non-synonymous nucleotide mutations are highlighted in cyan. The motifs of the active polymerase site are shown in red, light-blue, green, purple and dark-blue letters and identified as motifs I, A, B, C and D. The asterisks denote identical amino acids.
